# Polydopamine functionalized VEGF gene-activated 3D printed scaffolds for bone regeneration†

**DOI:** 10.1039/d1ra01193f

**Published:** 2021-04-08

**Authors:** Jaidev L. Chakka, Timothy Acri, Noah Z. Laird, Ling Zhong, Kyungsup Shin, Satheesh Elangovan, Aliasger K. Salem

**Affiliations:** Department of Pharmaceutics and Experimental Therapeutics, College of Pharmacy, University of Iowa Iowa City IA-52242 USA aliasger-salem@uiowa.edu +1-319-335-8810; Department of Experimental Research, Sun Yat-sen University Guangzhou PR China; Department of Orthodontics, College of Dentistry and Dental Clinics, University of Iowa Iowa City IA-52242 USA; Department of Periodontics, College of Dentistry and Dental Clinics, University of Iowa Iowa City IA-52242 USA

## Abstract

Bone is a highly vascularized organ and the formation of new blood vessels is essential to regenerate large critical bone defects. In this study, polylactic acid (PLA) scaffolds of 20–80% infill were three-dimensionally (3D) printed using a fused deposition modeling based 3D printer. The PLA scaffolds were coated with polydopamine (PDA) and then were surface-functionalized with polyethyleneimine (PEI) and VEGF-encoding plasmid DNA (pVEGF) nanoplexes (PLA-PDA-PEI-pVEGF). The PLA-PDA-PEI-pVEGF scaffolds with 40% infill demonstrated higher encapsulation efficiency and sustained release of pVEGF than scaffolds with 20, 60 and 80% infill and were therefore used for *in vitro* and *in vivo* studies. The PLA-PDA-PEI-pVEGF increased the translation and secretion of VEGF and BMP-2. The PLA-PDA-PEI-pVEGF also yielded a 2- and 4.5-fold change in *VEGF* and *osteocalcin* gene expression *in vitro*, respectively. A tube formation assay using human umbilical vascular endothelial cells (HUVECs) showed a significant increase in tube length when exposed to the PLA-PDA-PEI-pVEGF scaffold, in comparison to PLA and PLA-PDA scaffolds. The PLA-PDA-PEI-pVEGF scaffold in an *in vivo* rat calvarial critical bone defect model demonstrated 1.6-fold higher new bone formation compared to the PLA-PDA scaffold. H&E and Masson's trichrome staining of bone sections also revealed that the PLA-PDA-PEI-pVEGF scaffold facilitated the formation of more blood vessels in the newly formed bone compared to the PLA and PLA-PDA scaffold groups. Thus, PLA-PDA-PEI-pVEGF might be a potential 3D printed gene activated scaffold for bone regeneration in clinical situations.

## Abstract 2

Bone is a highly vascularized organ and the formation of new blood vessels is essential to regenerate large critical bone defects.

## Graphical abstract

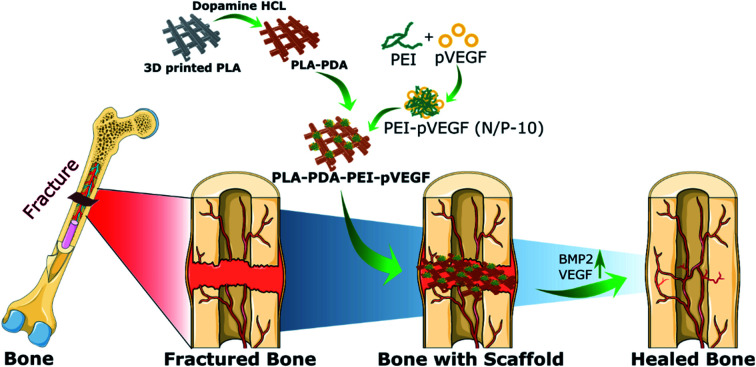

## 1. Introduction

Bone regeneration in large critical bone defects caused by injury or trauma is still a challenging therapeutic goal.^1,2^ The lack of vasculature is one significant reason for inadequate bone regeneration, leading to fracture and non-unions.^3–5^ VEGF is a unique mitogen factor that has a crucial role in angiogenesis-coupled bone repair and regeneration.^6,7^ Several approaches, including the administration of copper nanoparticles and the use of composite scaffolds, have been studied for their abilities to promote bone regeneration by enhancing angiogenesis.^8–11^ The therapeutic application of VEGF in protein form has disadvantages due to its instability, short half-life, and short-term effects.^12,13^ The immobilization of VEGF proteins by physical adsorption to delivery devices/implants leads to a burst release of proteins, which provides only short-term benefits.^13,14^ Sustained release of such therapeutic proteins is essential for predictable bone regeneration.^15^

Three-dimensional (3D) printing technology has paved the way for developing unique tissue engineering scaffolds that provide sustained release of osteogenic growth factors.^16,17^ Conventional autologous or allogeneic grafts are generic with little to no specificity with respect to bone defect morphology and dimensions that are unique to each patient.^18^ 3D printing has gained momentum in recent years due to the necessity of delivering unique personalized solutions to patient needs.^19–21^ The typical 3D printing workflow consists of developing a computer-aided design (CAD) model of the bone defect using clinically obtained computed tomography (CT) scans converting the CAD model to a .gcode file, and finally printing the planned model using a 3D printer.^18,22–24^ Fused deposition modeling (FDM) based 3D printing is the most popular rapid prototyping method used where a polymer filament is melted (>200 °C) and cast into a stack of 2D layers. This technique utilizes thermoplastic biodegradable and biocompatible polymers such as polylactic acid (PLA).^24^ PLA is a Food and Drug Administration cleared biocompatible polymer widely used in biomedical applications and has a broad scope for surface functionalization and modification.^24^ The acid end groups of PLA polymers render a negative charge to the scaffold further enhancing surface reactivity.^24^

For 3D printed scaffolds, in addition to surface properties, porosity will also play a significant role in influencing bone regeneration.^25^ PLA scaffolds with pore sizes similar to native bone (500 μm) have demonstrated optimal osteogenic differentiation of mesenchymal stem cells *in vitro*.^24,26^ In another study, calcium phosphate scaffolds with an average pore size of 400 μm showed increased ectopic bone formation in dogs, over scaffolds with pores of 200 μm diameter.^26^ Research thus far suggests that the pore size of a porous scaffold should be >300 μm in diameter in order to favor bone regeneration.^25,27,28^

Non-viral gene delivery is a cost-effective and safe approach facilitating stable delivery of desired genes to host cells.^29^ Non-viral gene delivery vectors are nanometer-sized complexes that can be prepared using cationic polymers such as polyethyleneimine (PEI), polyamidoamine (PAMAM), or chitosan complexed with anionic DNA or RNA.^30–35^ Our group has significant expertise in developing nanoplexes using branched polyethyleneimine (PEI) complexed with plasmid DNA (pDNA) or chemically modified RNA (cmRNA).^30,31,35–38^

For the past decade, 3D printed scaffolds have been studied for their application in bone regeneration.^39,40^ Progress was initially made as researchers elucidated the structural and mechanical features of the scaffolds using different materials produced with different 3D printing techniques.^40^ Subsequently, the bioactive potential of scaffolds became apparent when researchers explored the possibility of incorporating proteins into scaffolds *via* encapsulation or physical adsorption. It is well known that proteins and DNA degrade at or beyond 60 °C. The blending of such temperature-sensitive genetic material with high-temperature (>200 °C) thermoplastic polymers during the 3D printing process is a significant challenge and surface functionalization of the scaffolds is a suitable alternative. The surface coating of 3D printed scaffolds with a functionalization moiety that can anchor the genetic material to the scaffold is necessary. Polydopamine (PDA) is a nature-inspired biomaterial prepared by self-assembly of dopamine hydrochloride under base conditions.^41^ PDA has excellent potential to bind to a broad range of substances such as proteins, genetic material and nanoparticles.^42^ PDA was also observed to promote cell proliferation and osteogenic differentiation of human bone marrow-derived mesenchymal stem cells (hBMSCs) *in vitro*, which can facilitate *in vivo* bone regeneration.^31^

In this study, we fabricated 3D printed PLA scaffolds with different pore sizes. These PLA scaffolds were then coated with PDA and further surface functionalized with PEI-pVEGF nanoplexes to fabricate PLA-PDA-PEI-pVEGF scaffolds. *In vitro* cell proliferation and osteogenic potential of fabricated scaffolds were evaluated using hBMSCs and the *in vivo* bone regeneration potential was assessed using a rat calvarial bone defect model.

## 2. Materials and methods

All materials used were of analytical grade, and the chemicals used were without any modification.

### 2.1 Fabrication of 3D printed PLA-PDA scaffolds

A polylactic acid (PLA) filament of 1.75 mm was procured from Prusa Research (Czech Republic). A Prusa printer (I3MK3sMMU2.0s, Prusa Research, Czech Republic) was used to fabricate the 3D printed scaffolds. The CAD model of a 6.2 × 2 mm disc was prepared using Fusion 360 (Autodesk, USA) software. The CAD model was exported to Prusa Slic3r. The layer height was set at 0.2 mm with a print speed of 40 mm s^−1^ and a print temperature of 210 °C with a bed temperature of 60 °C. A range of infill percentages from 20, 40, 60, and 80% were used to fabricate scaffolds. The infill percentage determines the amount of polymer deposited during printing which leads to different pore sizes. The PLA scaffolds were printed and stored dry at room temperature. For PDA coating, the PLA scaffolds were immersed in 2 mg mL^−1^ dopamine chloride (Sigma Aldrich, USA) dissolved in Tris base of pH 8.5 for 2 h (PLA-PDA). The scaffolds were then air-dried and stored in a vacuum desiccator until further use.

### 2.2 Fabrication of PLA-PDA-PEI-pVEGF scaffolds

The VEGF plasmid (VEGFA (NM_001025366) Human Tagged ORF Clone, ∼4.9 kb, CMV promoter) was procured from Origene Technologies, Inc., USA and was transformed into the *E. coli* strain DH5α, and the bacteria were cultured in Luria broth media for 48 h in a shaking incubator at 37 °C at 300 rpm. The bacteria were pelleted *via* centrifugation at 8000 × *g* for 15 min (Eppendorf, USA). The pVEGF from the bacterial pellet was extracted using a plasmid extraction kit (Maxiprep, Sigma Aldrich) and the extracted plasmids were quantified at 260 nm using a spectrometer (Nanodrop, USA) and stored at −20 °C until further use.

The PLA-PDA scaffolds were sterilized under UV light for 1 h in a biosafety cabinet (Class II, Steriguard III Advance, The Baker Company, USA). The PEI-pVEGF nanoplexes were prepared by mixing appropriate volumes of cationic branched PEI and pDNA (N/P ratio = 10) under sterile conditions. The appropriate volume of nanoplexes equivalent to 5 μg pVEGF was added to each scaffold and incubated for 14 h in a CO_2_ incubator at 37 °C. The unbound nanoplexes were removed and stored for encapsulation efficiency measurements. The scaffolds were washed twice with sterile PBS and then used immediately for *in vitro* studies.

### 2.3 Characterization of scaffolds

The 3D printed PLA and PLA-PDA scaffolds with different infill percentages were imaged using a scanning electron microscope (FESEM, HITACHI S4500, HITACHI, Japan). The surface wettability was tested using a contact angle goniometer equipped with a motion capture camera (Ram-e-hart, Italy). Scaffolds with 100% infill were used for contact angle experiments where a 5 μL water droplet was placed on the scaffold and the image was processed using ImageJ contact angle plugin. The scaffolds' compressive strength was evaluated using a uniaxial tensile testing machine (Instron, USA) with 5 N of the load cell. The scaffolds were allowed to be compressed at 1 N min^−1^ until the compression reached half of the scaffolds' height.

### 2.4 Plasmid encapsulation and release

The pVEGF used in the encapsulation and release experiments was quantified using the PicoGreen™ assay (QuantiDNA, Thermofisher, USA). To determine the efficiency of complexation of pVEGF with PEI, PEI-pVEGF nanoplexes were disassociated with heparin. Briefly, 100 μL of nanoplexes containing 5 μg of pVEGF were incubated with 10 μL of heparin (1.2 mg mL^−1^) for 15 min. The pVEGF released from the nanoplexes was then quantified.

To assess pVEGF encapsulation on the scaffolds, the unbound nanoplexes were collected and incubated with 10 μL of heparin (1.2 mg mL^−1^) for 15 min. The pVEGF dissociated from the complexes were quantified.

For the pVEGF release study, PLA-PDA-PEI-pVEGF scaffolds were incubated in 200 μL of 1× PBS at pH 7.4 in a 37 °C incubator. Aliquots of a total of 200 μL were withdrawn initially every hour for 6 h and then once every 24 h until day 6. Fresh PBS of 200 μL was replenished every time the PBS was collected. The percentage release was calculated using the following formula.% pVEGF released = (cumulative amount of pVEGF released/initial amount of pVEGF encapsulated) × 100

The PBS samples were treated with 10 μL heparin (1.2 mg mL^−1^) to dissociate any PEI-pVEGF complexes. The dissociated pVEGF was quantified.

### 2.5 *In vitro* studies

Human bone marrow-derived mesenchymal stem cells (hBMSC) were commercially obtained (Lonza, USA) and had the following stem cell marker profile: CD105^+^, CD73^+^, CD45^−^ and CD34^−^. Cells were cultured in low glucose Dulbecco modified Eagle's medium supplemented with 10% fetal bovine serum (FBS), 1% sodium pyruvate and 1% penicillin–streptomycin. Cell proliferation of hBMSC on PLA, PLA-PDA, and PLA-PDA-PEI-pVEGF scaffolds was assessed by seeding 25 000 hBMSCs onto UV light-sterilized (1 h) scaffolds in 96 well plates. The scaffolds were incubated in a 5% CO_2_ incubator at 37 °C. After incubation, cell proliferation on scaffolds at days 3, 7, and 14 was analyzed using an MTS assay (Promega, USA). The optical density (OD) was read at 450 nm using a UV-Vis spectrophotometer (M5 Spectromax, Molecular Devices, USA).

After a 14 day incubation period, the cells were fixed with 3.7% formaldehyde for 30 min and washed with Milli-Q water. The cells were incubated sequentially with a range of ethanol solutions increasing from 20 to 100% for 15 min each. The final 100% ethanol solution was removed and then the cells were incubated with hydroxymethyldisilazane (HMDS, Sigma Aldrich, USA) overnight. After incubation, HMDS was removed and the scaffolds were air dried. Cells were then coated with palladium and silver using an ion sputter coater for 1 min and imaged using scanning electron microscopy (FESEM, HITACHI S4500, HITACHI, Japan). For ELISA, 25 000 hBMSCs were seeded onto the scaffolds and cultured for 7 days. The secretion of BMP-2 and VEGF in the cell supernatants was analyzed using ELISA as per the manufacturer's protocol (R&D Systems, USA). Expression of *VEGF* and *osteocalcin* genes was evaluated using Real-Time Polymerase Chain Reaction (RT-PCR) using the following primers (IDT, USA).

*VEGF*:

Forward-5′CTTCTGAGTTGCCCAGGAGA3′;

Reverse-5′CTCACACACACACAACCAGG3′

*Osteocalcin*:

Forward-5′TAGTGAAGAGACCCAGGCGC3′;

Reverse-5′CACAGTCCGGATTGAGCTCA3′

The hBMSCs (25 000 cells) were seeded onto the indicated scaffolds and incubated until day 7. The media was removed, and the cells were lysed using the RLT buffer (Qiagen, USA). Using the miniRNeasy extraction kit (Qiagen, USA), RNA was extracted from the cell lysate and was quantified using a Nanodrop spectrophotometer (Nanodrop, USA). RNA was then converted to cDNA using the cDNA conversion kit (Advanced Biosystems, USA). The cDNA was mixed with primers and a SYBR green reagent (SYBRfast, Advanced Biosystems, USA). The samples were analyzed using an RT-PCR machine (Applied Biosystems, USA) as per the manufacturer's protocol. The 2^−ΔΔ*C*_t_^ values were obtained, and the fold changes were reported.

Alkaline phosphatase expression and Alizarin red staining was carried out by first incubating 25 000 hBMSCs onto a scaffold and incubating them for 14 days. For the alkaline phosphatase assay, the spent media was removed, and cells were washed with PBS. A cell lysis solution of 0.1% Triton X-100 was added to each well and incubated for 15 min. Then 100 μL of cell lysate was mixed with 100 μL of poly nitrophenyl phosphate (Sigma Aldrich) solution and incubated in the dark for 1 h. The reaction was stopped with 0.1 M NaOH solution (Sigma Aldrich), and the wells were read at 410 nm using a UV-Vis spectrophotometer (M5 Spectromax, Molecular Devices). For Alizarin red staining, after 14 days of incubation, the spent media was removed and the scaffolds were treated with 3.7% formaldehyde solution (Sigma Aldrich) for 30 min. The scaffolds were washed with PBS and then incubated with 2% Alizarin red S solution (Sigma Aldrich) for 30 min. The excess dye was removed, and the scaffolds were washed four times with copious amounts of Milli-Q water. The scaffolds were air-dried, and then 100 μL of 0.5 M HCl + 0.5% SDS solution was added to each well and incubated for 15 min. The dissolved dye in the solution was read at 410 nm using a UV-Vis spectrophotometer (M5 Spectromax, Molecular Devices).

### 2.6 Tube formation assay

Human Umbilical Vascular Endothelial Cells (HUVECs) were procured from ATCC, USA. The cells were cultured in endothelial complete growth media (EGM, ATCC) and incubated in a 5% CO_2_ incubator at 37 °C. The PLA, PLA-PDA and PLA-PDA-PEI-pVEGF scaffolds were UV sterilized and added to wells of 96 well plates. The scaffolds were incubated in serum-free EGM media for 24 h. The supernatant was collected (referred to as conditioned media) and used the same day for the assay. Matrigel (BD Biosciences, USA) was thawed at 4 °C overnight and 50 μL was added into each well of a 96 well plate. The plate was incubated in a 5% CO_2_ incubator at 37 °C for 30 min. HUVECs (5 × 10^4^) mixed with 200 μL of conditioned media were added onto the gelated Matrigel and incubated for 6 h. The EGM media with serum supplement was used as a control. After 6 h incubation, the brightfield images of the network formed by the HUVECs on Matrigel were taken for all the groups. The tube length of the tube network formed by the HUVECs in each group was calculated using Angiogenesis Analyzer plugin in ImageJ (ImageJ 1.51j8, USA).

### 2.7 *In vivo* studies

Animal studies were performed with prior approval from the University of Iowa Institutional Animal Care and Use Committee (Protocol #8102185). Male Fisher 344 rats (Charles River, Wilmington, MA) at 14 weeks of age were used for this study. All animals were housed in animal care facilities and cared for, according to the guidelines established by the University of Iowa Institutional Animal Care and Use Committee. Before the survival surgeries, the animals were anesthetized by intraperitoneal injection of 95 mg kg^−1^ ketamine and 13.5 mg kg^−1^ xylazine mixture solution. Meloxicam (Boehringer Ingelheim, Rhein, Germany) was also administered before beginning the surgery. To gain access to the calvaria, incisions were made through the skin and the periosteum, and the soft tissue was reflected using a flat blade to expose the bone. On either side of the sagittal suture, a 5 mm diameter defect was created in the calvaria using a 5 mm trephine burr. For this study, printed scaffolds of 5 mm diameter and 1 mm height were utilized. For the *in vivo* study, three groups were included: empty defect (*N* = 5); PLA-PDA (*N* = 5); and PLA-PDA-PEI-pVEGF (*N* = 5). The respective scaffolds were randomly implanted into the defect sites, and the periosteum was sutured over the implants to minimize the movement of the implants. This was followed by suturing the overlying skin. After four weeks, the animals were euthanized, and the bone samples with defects were extracted and fixed in 10% neutral buffered formalin for analysis by micro-computed tomography (micro-CT) analysis and histology.

A Skyscan 1272, a high-resolution 3D X-ray microscope (Bruker, Billerica, MA), set at 100 kV and 142 μA was used to scan the defects with a voxel size of 21.7 μm^3^. A stringent 3.5 mm diameter region of interest (ROI) was used to assess new bone volume. Bone tissue was identified based on a density threshold, and bone volume per total volume was calculated using Dragon Fly analysis software (Object Research Systems, Quebec, Canada).

After micro-CT analysis, the samples were incubated in the ethylene diamine tetra-acetic acid (EDTA) solution to decalcify tissues for two weeks. The decalcified solution was replaced every two days. The decalcified solution was assessed for calcium concentration by adding ammonium oxalate. The tissue was determined to be fully decalcified when no precipitates formed. Each defect was dissected so that the tissue slice ran through the center of the defect in question. The decalcified tissue was paraffin-embedded, processed with a microtome, mounted and stained with hematoxylin and eosin (H&E) and Masson's trichrome stain. The brightfield images were captured using an inverted microscope (Olympus BX61, USA) equipped with camera (Olympus, USA) and new bone formation was assessed histologically.

### 2.8 Data analysis

For all the *in vitro* experiments in this study, we had at least three samples in every group at any given time point, and the data was represented in mean ± standard deviation. To assess differences between groups, mean values, one-way analysis of variance (ANOVA) coupled with Tukey's post-test was performed. Significant differences are noted as *p < 0.05, **p < 0.01, ***p < 0.005 and ****p < 0.001.

## 3. Results

### 3.1 Characterization of PEI-pVEGF nanoplexes and 3D printed scaffolds

**Fig. 1:**
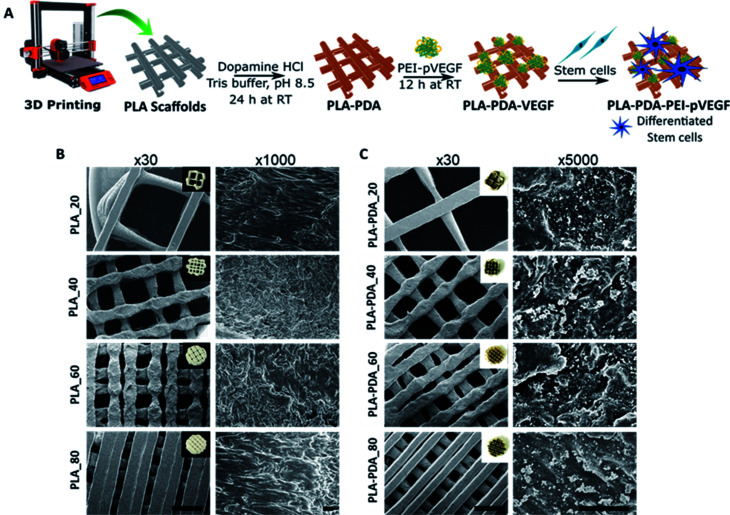
Fabrication of PDA functionalized 3D printed PLA scaffolds coated with PEI-pVEGF. Schematic showing the overall process of generating functionalized 3D printed scaffolds and *in vitro* testing of scaffolds using hBMSCs (A). SEM images of 3D printed PLA scaffolds with 20, 40, 60 and 80% infill at 30× (scale bar is 1 mm) and 1000× (scale bar is 50 μm) magnifications along with insets showing optical images of printed scaffolds (B). SEM images of the PDA functionalized 3D printed PLA scaffolds with 20, 40, 60 and 80% infill at 30× (scale bar is 1 mm) and 5000× (scale bar is 50 μm) magnifications along with and insets showings the optical images of printed scaffolds (C). RT in (A) denotes room temperature.

The PEI-pVEGF nanoplexes (N/P ratio of 10) had a mean particle size of 288 ± 14 nm and a mean zeta potential of 33 ± 1 mV. Fig. 1A shows a schematic of the fabrication of a 3D printed PLA scaffold surface functionalized with PDA and PEI-pVEGF to form a PLA-PDA-PEI-pVEGF scaffold and the experimental setup to assess *in vitro* osteogenicity. Fig. 1B and C shows SEM micrographs of the PLA and PLA-PDA scaffolds with different infill percentages at low (30×) and high (1000× & 5000×) magnification. The inset shows the optical images of the scaffolds with and without PDA modification. The PDA coating had changed the color of the PLA scaffolds from white to brownish. The 30× images show the reduction in pore size with increasing infill percentages. The pore diameter was evaluated using ImageJ and is shown in Table S1.† The PLA_20 scaffolds had the largest pore size (1888 ± 68 μm), whereas the PLA_80 scaffolds had the smallest (222 ± 78 μm). The PDA coating did not affect the pore size of the scaffolds and the pore size of the PLA_40 and PLA-PDA_40 scaffolds is similar to that of trabecular bone (∼500 μm). The 5000× magnification images show the PDA aggregates coated onto the surface of the PLA-PDA scaffolds (Fig. 1C).

### 3.2 Encapsulation and release of pVEGF

The complexation of pVEGF in the PEI-pVEGF nanoplexes was 4.455 μg for a given 5 μg. Table 1 shows the encapsulation efficiencies of PEI-pVEGF nanoplexes among the fabricated scaffolds with different infill percentages. The PLA-PDA-PEI-pVEGF_40 scaffolds with the average pore diameter closer to that of trabecular bone (∼500 μm) showed higher encapsulation efficiency when compared to PLA-PDA-PEI-pVEGF scaffolds with 20, 60, and 80% infill.

**Table 1:** pVEGF encapsulation efficiency in 3D printed scaffolds and cumulative release measurements of pVEGF (over 144 hours)

Scaffold	Encapsulation of pVEGF (μg)	Encapsulation efficiency (%)	Cumulative release of pVEGF (μg)	Cumulative release of pVEGF (%)
PLA-PDA-PEI-pVEGF_20	0.478 ± 0.008	10	0.447 ± 0.005	93
PLA-PDA-PEI-pVEGF_40	2.716 ± 0.273	60	0.420 ± 0.001	15
PLA-PDA-PEI-pVEGF_60	1.551 ± 0.068	34	0.442 ± 0.004	28
PLA-PDA-PEI-pVEGF_80	1.638 ± 0.210	36	0.418 ± 0.003	25

**Fig. 2:**
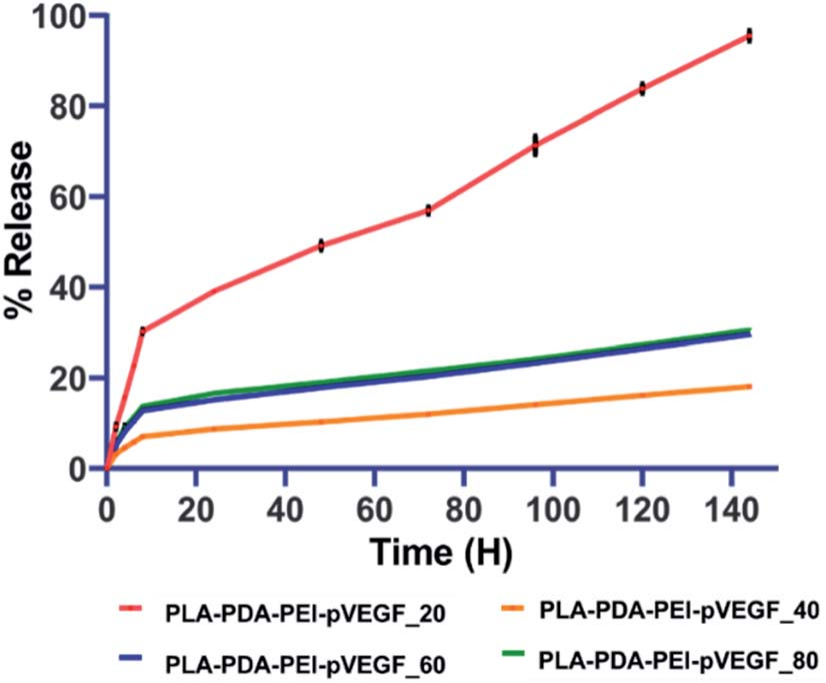
Release of pVEGF from indicated PLA-PDA-PEI-pVEGF scaffolds with 20, 40, 60 and 80% infill. Three samples per group per time point were used for this release kinetics study and the data are presented as mean ± standard deviation.

In all the groups tested, pVEGF release studies (Fig. 2) showed an initial burst release pattern for the first 6 h, followed by a sustained release from the scaffolds over the next 6 days. The PLA-PDA-PEI-pVEGF_20 scaffolds released pVEGF faster and close to 100% release was noted by day 6. In contrast, PLA-PDA-PEI-pVEGF_40, 60 and 80 scaffolds displayed a lower burst release and slower release of pVEGF. Of these scaffolds, pVEGF release was slowest from PLA-PDA-PEI-pVEGF_40 scaffolds with only fifteen percent of pVEGF released in 144 h. The kinetics of the pVEGF release was analyzed using standard mathematical models, as provided in Table S2.† Based on the porosity, higher encapsulation and the ability to release pVEGF in a sustained manner, PLA-PDA-PEI-pVEGF_40 scaffolds were selected to be used for the rest of our *in vitro* experiments and for our *in vivo* experiments. For the sake of simplicity, PLA-PDA-PEI-pVEGF_40 scaffolds will be hereafter referred to as PLA-PDA-PEI-pVEGF.

### 3.3 *In vitro* cell attachment and cell proliferation assays

**Fig. 3:**
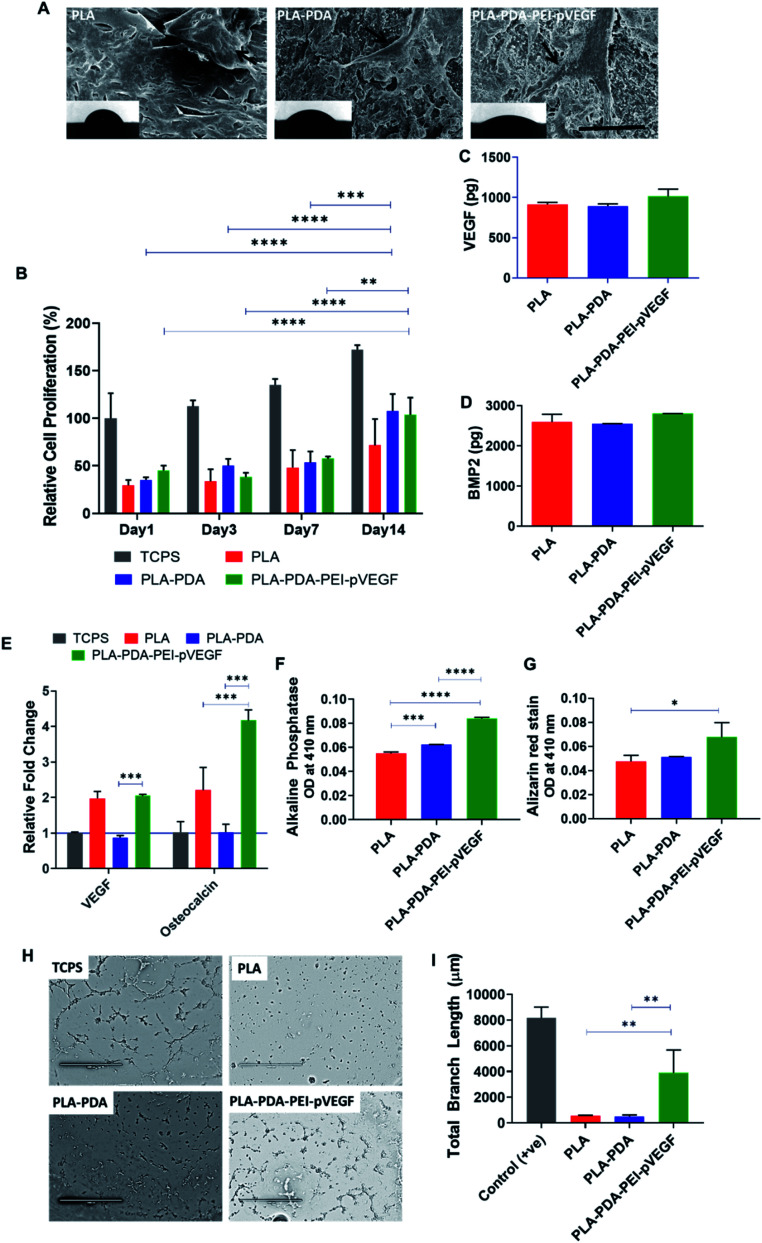
*In vitro* cellular studies. SEM micrographs (scale bar is 30 μm) showing hBMSCs attachment on the scaffold surface and the inset show the contact angle goniometer images of the scaffolds, respectively (A). Cell proliferation of hBMSCs on indicated surfaces, TCPS denotes tissue culture polystyrene surface used as a control and significant differences were observed for groups PLA-PDA and PLA-PDA-PEI-pVEGF over time (as indicated) (B). Color-coded legend also applies to figures (C–G) & (I). ELISA measuring VEGF (C) and BMP-2 (D) release from hBMSCs cultured on indicated scaffolds on day 7. RT-PCR study showing the relative levels of expression of VEGF and osteocalcin mRNA and significant differences were observed between PLA-PDA and PLA-PDA-PEI-pVEGF groups in expression of *VEGF* and *osteocalcin* genes (as indicated) (E). Alkaline phosphatase assay showing significant differences in activity between PLA, PLA-PDA and PLA-PDA-PEI-pVEGF groups (F) and Alizarin red staining of hBMSCs showing significant mineralization between PLA and PLA-PDA-PEI-pVEGF groups (G) cultured on indicated scaffolds on day 7. Brightfield microscopy (scale bar is 1 mm) images of the tube formation assay using HUVECS after 4 h (H) and quantified total branch length from the brightfield images of the tube formation assay using ImageJ. Significant differences in the total branch length were observed between PLA *vs.* PLA-PDA-PEI-pVEGF and PLA-PDA *vs.* PLA-PDA-PEI-pVEGF groups (I). The data are represented as mean ± standard deviation for three repeats. The significant differences were assessed by one-way ANOVA with *p < 0.05, **p < 0.01, ***p < 0.005 and ****p < 0.001.

The cell attachment of hBMSCs on PLA, PLA-PDA, and PLA-PDA-PEI-pVEGF scaffolds was shown in Fig. 3A. The black arrows on the SEM images show the attachment of cells to the scaffold. The insets show the contact angle images validating the hydrophilicity of the scaffolds where the contact angles for the PLA, PLA-PDA, and PLA-PDA-PEI-pVEGF scaffolds were 62 ± 5°, 41 ± 2°, and 32 ± 4°, respectively.

*In vitro* cell proliferation of hBMSCs on different scaffolds was assessed using an MTS assay (Fig. 3B). The data showed a significant increase in cell proliferation on PLA-PDA and PLA-PDA-PEI-pVEGF by day 14 compared to day 1, 3, & 7. By day 14, the cell proliferation in PLA-PDA and PLA-PDA-PEI-pVEGF is comparatively higher than PLA scaffolds.

### 3.4 Evaluation of *in vitro* osteogenic potential of scaffolds

The secretion of VEGF and BMP-2 proteins by hBMSCs cultured on the three different scaffolds was assessed using ELISA with no statistical difference observed between the groups (Fig. 3C and D). However, cells cultured on PLA-PDA-PEI-pVEGF exhibited a marginal increase in VEGF and BMP-2 secretion by day 7.

The RT-PCR analysis (Fig. 3E) revealed a higher fold expression of *VEGF* and *osteocalcin* genes in cells cultured on PLA-PDA-PEI-pVEGF scaffolds compared to the tissue culture polystyrene surface (TCPS) control. VEGF mRNA expressed in cells cultured on the PLA scaffolds, and PLA-PDA-PEI-pVEGF were close to 2-fold higher than in cells grown on PLA-PDA scaffolds. However, the cells cultured on PLA-PDA-PEI-pVEGF had a 4-fold higher expression of *osteocalcin* genes than in cells grown on TCPS or PLA-PDA scaffolds, indicating a potential role of VEGF in inducing this genetic marker for osteogenic differentiation.^43^ PLA-PDA-PEI-VEGF promoted significantly higher expression of alkaline phosphatase by hBMSCs compared to PLA and PLA-PDA scaffolds (Fig. 3F). Alizarin red staining, a marker to determine calcium phosphate mineralization by hBMSCs, showed significantly higher mineralization by the cells exposed to PLA-PDA-PEI-pVEGF than by cells exposed to PLA scaffolds (Fig. 3G).

The tube formation assay results in Fig. 3H showed that the branching of the endothelial cell vascular network was higher in the PLA-PDA-PEI-pVEGF group compared to PLA or PLA-PDA scaffold groups (Fig. 3I).

### 3.5 *In vivo* bone regeneration in a rat calvarial defect model

**Fig. 4:**
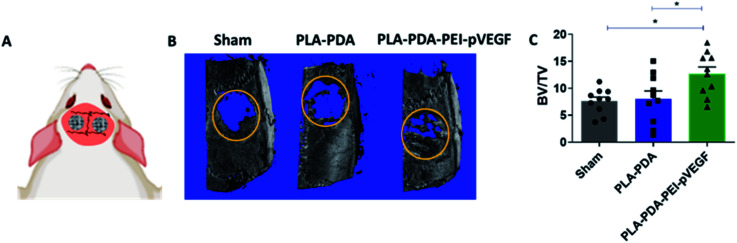
Bone regeneration in a rat calvarial defect model. A schematic showing bilateral rat calvarial defects treated with the scaffolds (A). Micro-CT scans showing regenerated bone within ROI (yellow circle) at day 30 post-implantation of indicated scaffold (B), and BV/TV in the three groups calculated using micro-CT images. Significant differences were observed between sham *vs.* PLA-PDA-PEI-pVEGF and PLA-PDA *vs.* PLA-PDA-PEI-pVEGF groups (C). The data were presented as mean ± standard deviation, with *N* = 10 and *p < 0.05.

Fig. 4B shows three-dimensional micro-CT scan images of rat calvarial defects 4 weeks after treatment with indicated scaffold, with sham (empty defect) surgery being the control. Defects treated with PLA-PDA-pVEGF showed significantly more new bone formation than the other groups tested (Fig. 4B and C). Fig. 4C shows the calculated bone volume/total volume (BV/TV) from the micro-CT images of the three groups tested.

**Fig. 5:**
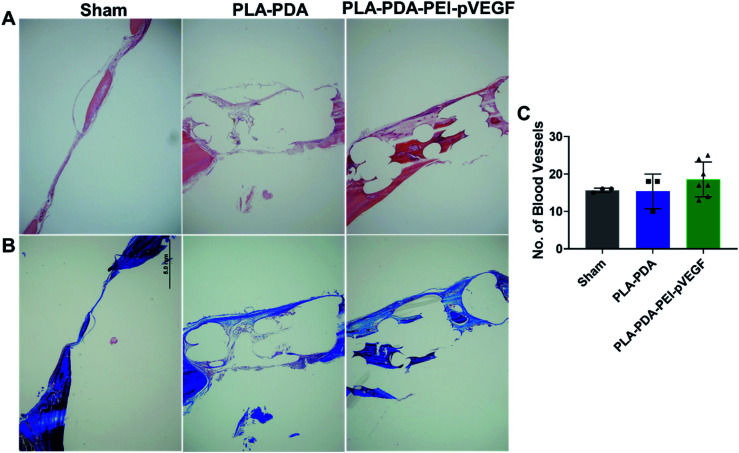
Histology of bone defects with indicated scaffolds on day 30. H&E staining (A) and Masson's trichrome staining (B) of the defect sites implanted with sham, PLA-PDA scaffolds, and PLA-PDA-PEI-pVEGF. Blood vessel number enumerated from the H&E stained images from sham, PLA-PDA scaffold, and PLA-PDA-PEI-pVEGF treated groups (C). The data are presented as mean ± standard deviation.

Representative H&E stained sections from the defects representing the three scaffold groups are shown in Fig. 5A. The data showed that new bone (light pink color in H&E images) formed (within the defect) across all scaffold groups. However, more light pink areas confirm more new bone tissue formation occurred when PLA-PDA-PEI-pVEGF was used compared to the other two groups. Masson's trichrome staining (Fig. 5B) further confirmed the H&E findings that more new bone tissue (light blue color) formation occurred in the PLA-PDA-PEI-pVEGF treated group, compared to the sham group. The formation of mature bone (dark blue color) was also more significant in the PLA-PDA-PEI-pVEGF treated group than in the PLA-PDA scaffold treated group. The number of blood vessels enumerated from the histological images indicated a marginally greater number of blood vessels in the defects treated with PLA-PDA-PEI-pVEGF *versus* PLA-PDA scaffold and sham control groups.

## 4. Discussion

Three-dimensional (3D) printing facilitates the fabrication of scaffolds with well-defined pore sizes (and interconnected pores) and a comparatively higher mechanical strength than porous scaffolds prepared by the traditional salt-leaching method.^44^ The sustained delivery of genes is essential for predictable bone regeneration over long time periods *in vivo*.^45^ The PLA-PDA-PEI-pVEGF scaffolds with 40% infill showed the slowest sustained release profile (over 144 hours) compared to scaffolds with 20, 60 and 80% infill (Fig. 2). The pore size, which is a direct reflection of the infill percentage, is an essential factor determining the encapsulation and release of pVEGF. The PLA-PDA-PEI-pVEGF_20 with larger pore size and PLA-PDA-PEI-pVEGF_80 with smaller pore size yielded lower encapsulation efficiencies (Table 1) compared to PLA-PDA-PEI-pVEGF_40. There was no significant change in the amount of pVEGF released from scaffolds with different pore sizes. This was due to the difference in the encapsulation efficiency of pVEGF between scaffolds with varying pore sizes.

A pore size similar to or closer to native bone has been proven optimal for bone regeneration.^46^ The PLA-PDA-PEI-pVEGF_40 scaffold had a pore size closer to that of bone and showed a higher encapsulation and slow-release, facilitating better bone formation *in vivo*. The optimal porosity also facilitates cell proliferation by maintaining an efficient oxygen and nutrient supply to the cells.^47^

PDA is an amine-rich, self-polymerizable material that can bind to a broad spectrum of biomolecules.^42^Fig. 3A shows that the PLA scaffolds with PDA coating and further functionalization with PEI-pVEGF have significantly reduced the contact angle compared to PLA alone. The lower contact angle with higher hydrophilicity also provides improved cell attachment.^48^ The hBMSCs attached on PLA are circular while, the hBMSCs on the PLA-PDA and PLA-PDA-PEI-pVEGF scaffolds are attached and well spread on the surface as indicated by the black arrows in Fig. 3A. The hBMSCs on the PLA-PDA-PEI-pVEGF scaffold also showed numerous filopodia, indicating favorable cell–cell and cell–matrix interactions.^24^

The sustained release of pVEGF from PLA-PDA-PEI-pVEGF showed a marginal increase in VEGF and BMP-2 secretion as determined by ELISA (Fig. 3C and D). Cells exposed to PLA-PDA-PEI-pVEGF scaffolds showed a significant expression by the *osteocalcin* gene, compared to PLA and PLA-PDA scaffolds (Fig. 3E) and significant bone mineralization (Fig. 3G). These results infer that the increased VEGF secretion had facilitated the production of BMP-2 from the hBMSCs leading to its differentiation towards osteoblasts as indicated by *osteocalcin* expression. For bone regeneration, VEGF uses two mechanisms: (1) VEGF secreted by osteoblast progenitor cells promotes osteoblast maturation and mineralization, and (2) VEGF secreted by osteoblast progenitors binds to the adjacent endothelial cells promoting neovascularization and thus enhances the expression of BMP-2 and BMP-4, both being potent osteogenic growth factors.^49–52^

The rat calvarial defect *in vivo* study showed that PLA-PDA-PEI-pVEGF delivered an increased bone volume (BV/TV) with significant new bone formation compared to PLA and PLA-PDA. The H&E and Masson Trichrome staining data showed the increased vasculature and new bone regions in PLA-PDA-PEI-pVEGF compared to PLA and PLA-PDA scaffold treated groups. Our *in vitro* findings correlated well with our *in vivo* results that highlighted the superiority of PLA-PDA-PEI-pVEGF scaffolds as a potential candidate for bone regeneration.

## 5. Conclusion

The 3D printed PLA-PDA-PEI-pVEGF scaffold with optimized pore size showed robust encapsulation and sustained VEGF release, leading to enhanced hBMSC proliferation and osteogenic differentiation *in vitro*. The cells cultured with PLA-PDA-PEI-pVEGF scaffolds produced increased amounts of BMP-2 and VEGF, stimulating angiogenesis, as evident from the tube formation assay. *In vivo*, implantation of PLA-PDA-PEI-pVEGF scaffolds resulted in significantly greater bone regeneration as measured by BV/TV, indicating our test scaffold to be a potential candidate in personalized bone regeneration applications that require further testing.

## Conflicts of interest

There are no conflicts to declare.

## Supplementary Material

RA-011-D1RA01193F-s001

## References

[cit1] Balogh Z. J., Reumann M. K., Gruen R. L., Mayer-Kuckuk P., Schuetz M. A., Harris I. A., Gabbe B. J., Bhandari M. (2012). Lancet.

[cit2] Cancedda R., Giannoni P., Mastrogiacomo M. (2007). Biomaterials.

[cit3] Lu C., Xing Z., Yu Y. y., Colnot C., Miclau T., Marcucio R. S. (2010). J. Orthop. Res..

[cit4] Mercado-Pagán Á. E., Stahl A. M., Shanjani Y., Yang Y. (2015). Ann. Biomed. Eng..

[cit5] Rouwkema J., Westerweel P. E., De Boer J., Verhaar M. C., Van Blitterswijk C. A. (2009). Tissue Eng., Part A.

[cit6] Hu K., Olsen B. R. (2016). Bone.

[cit7] Hu K., Olsen B. R. (2016). J. Clin. Invest..

[cit8] Clark D., Wang X., Chang S., Czajka-Jakubowska A., Clarkson B., Liu J. (2015). J. Biomed. Mater. Res., Part A.

[cit9] Street J., Bao M., deGuzman L., Bunting S., Peale F. V., Ferrara N., Steinmetz H., Hoeffel J., Cleland J. L., Daugherty A. (2002). Proc. Natl. Acad. Sci. U. S. A..

[cit10] Poldervaart M. T., Gremmels H., van Deventer K., Fledderus J. O., Öner F. C., Verhaar M. C., Dhert W. J., Alblas J. (2014). J. Controlled Release.

[cit11] Liu H., Du Y., Yang G., Hu X., Wang L., Liu B., Wang J., Zhang S. (2020). Adv. Healthcare Mater..

[cit12] Ma C., Jing Y., Sun H., Liu X. (2015). Adv. Healthcare Mater..

[cit13] Wu L., Gu Y., Liu L., Tang J., Mao J., Xi K., Jiang Z., Zhou Y., Xu Y., Deng L. (2020). Biomaterials.

[cit14] Borselli C., Ungaro F., Oliviero O., d'Angelo I., Quaglia F., La Rotonda M. I., Netti P. A. (2010). J. Biomed. Mater. Res., Part A.

[cit15] Shen X., Zhang Y., Gu Y., Xu Y., Liu Y., Li B., Chen L. (2016). Biomaterials.

[cit16] Langer R. (1997). Pharm. Res..

[cit17] Laurencin C. T., Khan Y. (2012). Sci. Trans. Med..

[cit18] Jang J., Park J. Y., Gao G., Cho D.-W. (2018). Biomaterials.

[cit19] Zhang L., Yang G., Johnson B. N., Jia X. (2019). Acta Biomater..

[cit20] Ashammakhi N., Hasan A., Kaarela O., Byambaa B., Sheikhi A., Gaharwar A. K., Khademhosseini A. (2019). Adv. Healthcare Mater..

[cit21] Do A. V., Khorsand B., Geary S. M., Salem A. K. (2015). Adv. Healthcare Mater..

[cit22] Arrigoni C., Gilardi M., Bersini S., Candrian C., Moretti M. (2017). Stem Cell Rev. Rep..

[cit23] Marolt D., Campos I. M., Bhumiratana S., Koren A., Petridis P., Zhang G., Spitalnik P. F., Grayson W. L., Vunjak-Novakovic G. (2012). Proc. Natl. Acad. Sci. U. S. A..

[cit24] Jaidev L., Chatterjee K. (2019). Mater. Des..

[cit25] Karageorgiou V., Kaplan D. (2005). Biomaterials.

[cit26] Yuan H., Kurashina K., de Bruijn J. D., Li Y., De Groot K., Zhang X. (1999). Biomaterials.

[cit27] Tsuruga E., Takita H., Itoh H., Wakisaka Y., Kuboki Y. (1997). J. Biochem..

[cit28] Kuboki Y., Jin Q., Kikuchi M., Mamood J., Takita H. (2002). Connect. Tissue Res..

[cit29] Shapiro G., Lieber R., Gazit D., Pelled G. (2018). Current Osteoporosis Reports.

[cit30] Atluri K., Seabold D., Hong L., Elangovan S., Salem A. K. (2015). Mol. Pharmaceutics.

[cit31] Chakka L. R. J., Vislisel J., Vidal C. d. M. P., Biz M. T., Cavalcanti B. (2020). Clinical Oral Investigations.

[cit32] Li J., Liang H., Liu J., Wang Z. (2018). Int. J. Pharm..

[cit33] Intra J., Salem A. K. (2010). J. Pharm. Sci..

[cit34] Rajasekaran D., Srivastava J., Ebeid K., Gredler R., Akiel M., Jariwala N., Robertson C. L., Shen X.-N., Siddiq A., Fisher P. B. (2015). Bioconjugate Chem..

[cit35] Khorsand B., Elangovan S., Hong L., Dewerth A., Kormann M. S., Salem A. K. (2017). AAPS J..

[cit36] Acri T. M., Laird N. Z., Jaidev L. R., Meyerholz D. K., Salem A. K., Shin K. (2020). Tissue Eng., Part A.

[cit37] Laird N. Z., Malkawi W. I., Chakka J. L., Acri T. M., Elangovan S., Salem A. K. (2020). J. Tissue Eng. Regener. Med..

[cit38] Elangovan S., Khorsand B., Do A.-V., Hong L., Dewerth A., Kormann M., Ross R. D., Sumner D. R., Allamargot C., Salem A. K. (2015). J. Controlled Release.

[cit39] Feng Y., Zhu S., Mei D., Li J., Zhang J., Yang S., Guan S. (2020). Curr. Drug Delivery.

[cit40] Ji K., Wang Y., Wei Q., Zhang K., Jiang A., Rao Y., Cai X. (2018). Bio-Des. Manuf..

[cit41] Batul R., Tamanna T., Khaliq A., Yu A. (2017). Biomater. Sci..

[cit42] Lynge M. E., van der Westen R., Postma A., Städler B. (2011). Nanoscale.

[cit43] Leach J. K., Kaigler D., Wang Z., Krebsbach P. H., Mooney D. J. (2006). Biomaterials.

[cit44] Zhao H., Li L., Ding S., Liu C., Ai J. (2018). Mater. Lett..

[cit45] Wang X., Hélary C., Coradin T. (2015). ACS Appl. Mater. Interfaces.

[cit46] Lee D. J., Kwon J., Kim Y. I., Wang X., Wu T. J., Lee Y. T., Kim S., Miguez P., Ko C. C. (2019). Orthodontics & Craniofacial Research.

[cit47] Yunoki S., Marukawa E., Ikoma T., Sotome S., Fan H., Zhang X., Shinomiya K., Tanaka J. (2007). J. Mater. Sci.: Mater. Med..

[cit48] Arima Y., Iwata H. (2007). Biomaterials.

[cit49] Thi M. M., Iacobas D. A., Iacobas S., Spray D. C. (2007). Ann. N. Y. Acad. Sci..

[cit50] Thi M. M., Suadicani S. O., Spray D. C. (2010). J. Biol. Chem..

[cit51] Schipani E., Maes C., Carmeliet G., Semenza G. L. (2009). J. Bone Miner. Res..

[cit52] Pearson H. B., Mason D. E., Kegelman C. D., Zhao L., Dawahare J. H., Kacena M. A., Boerckel J. D. (2019). Tissue Eng., Part A.

