# Supplementary material for: Polydopamine functionalized VEGF gene-activated 3D printed scaffolds for bone regeneration
Source: RSC Adv. 2021 Apr 8;11(22):13282–91. doi: 10.1039/d1ra01193f (PMC8697638; doi:10.1039/d1ra01193f)
Supplement: RA-011-D1RA01193F-s001 [file RA-011-D1RA01193F-s001.pdf]

Electronic Supplementary Material (ESI) for RSC Advances.
This journal is © The Royal Society of Chemistry 2021

Supplementary Information

Polydopamine mediated Vascular Endothelial Growth Factor Gene Activated Three-Dimensional Printed Scaffolds for Bone Regeneration

Jaidev L. Chakka[1], Timothy Acri[1], Laird Z. Noah[1], Ling Zhong[2], Kyungsup Shin[3], Satheesh Elangovan[4], and Aliasger K. Salem[1*]

[1]*Department of Pharmaceutics and Experimental Therapeutics, College of Pharmacy, University of Iowa, Iowa City, IA-52242, USA*

[2]*China*

[3]*Department of Orthodontics, College of Dentistry and Dental Clinics, University of Iowa, Iowa City, IA-52242, USA*

[4]*Department of Periodontics, College of Dentistry and Dental Clinics, University of Iowa, Iowa City, IA-52242, USA*

Table S1: Pore sizes of 3D printed PLA and PLA-PDA scaffolds with 20, 40, 60 & 80 % infill

**Scaffold**	**Average Pore size (µm)**
PLA_20	1888±68
PLA_40	673±183
PLA_60	362±141
PLA_80	222±78
PLA-PDA_20	1748±196
PLA-PDA_40	689±140
PLA-PDA_60	375±128
PLA-PDA_80	224±84

Table S2: Drug Release kinetics of the pVEGF released from the PLA-PDA-PEI-pVEGF fitting (R^2 values) with standard mathematical models

**No.**	**Scaffold group**	**Zero Order**	**First Order**	**Higuchi**	**Korsmeyer Peppas**	**Hopfenberg**	**Baker-Lonsdale**	**Weibull**
1	PLA-PDA-PEI-pVEGF_20	0.0939	0.6823	0.8280	0.9712	0.5690	0.6823	0.9269
2	PLA-PDA-PEI-pVEGF_40	0.2332	0.2883	0.8684	0.9712	0.2703	0.2880	0.8788
3	PLA-PDA-PEI-pVEGF_60	0.4129	0.5005	0.9178	0.9746	0.4729	0.5003	0.9303
4	PLA-PDA-PEI-pVEGF_80	0.7491	0.7892	0.9747	0.9756	0.7767	0.7891	0.9741
